# Optimal Linear Filter Based on Feedback Structure for Sensing Network with Correlated Noises and Data Packet Dropout

**DOI:** 10.3390/s23125673

**Published:** 2023-06-17

**Authors:** Weichen Shang, Hang Yu, Qingyu Li, He Zhang, Keren Dai

**Affiliations:** 1 School of Mechanical Engineering, Nanjing University of Science and Technology, Nanjing 210018, China; swc19960512@njust.edu.cn (W.S.); hangyu@njust.edu.cn (H.Y.); 2 North Information Control Research Academy Group Co., Ltd., Nanjing 211153, China; liqy14@tsinghua.org.cn

**Keywords:** distributed sensing, correlated noise, feedback structure, packet dropout

## Abstract

This paper is concerned with the estimation of correlated noise and packet dropout for information fusion in distributed sensing networks. By studying the problem of the correlation of correlated noise in sensor network information fusion, a matrix weight fusion method with a feedback structure is proposed to deal with the interrelationship between multi-sensor measurement noise and estimation noise, and the method can achieve optimal estimation in the sense of linear minimum variance. Based on this, a method is proposed using a predictor with a feedback structure to compensate for the current state quantity to deal with packet dropout that occurs during multi-sensor information fusion, which can reduce the covariance of the fusion results. Simulation results show that the algorithm can solve the problem of information fusion noise correlation and packet dropout in sensor networks, and effectively reduce the fusion covariance with feedback.

## 1. Introduction

Unmanned aerial vehicles (UAVs) have received a lot of attention in recent years. UAVs have experienced rapid growth due to their widespread use in the military industry [1,2,3] and the civilian world. However, as the battlefield environment faced by UAVs becomes more and more complex, individual UAVs face limitations in reconnaissance angles and destruction capabilities when performing missions such as reconnaissance or attack, and it is becoming increasingly difficult for individual UAVs to complete their missions, so the trend [4] has been for multiple UAVs to collaborate on combat technology. UAVs participate in collaborative operations in which sensors collect and acquire a large amount of battlefield situational data, carry out information fusion, collaborative search elements, target assignment, conduct coordinated operations, and use various airborne weapons to achieve strikes against enemy targets and complete combat missions. As the size and number of UAVs increase, unreliable communication links between sensors, random time lags and packet dropout (or uncertain observations) are common in the data transmission of real network systems. On the other hand, accurate or perfect information about the system model is usually not available in practice, so uncertainty is inevitable in UAV navigation and positioning. Therefore, the navigation and positioning technology based on the fusion of multi-sensor data has become a research hot spot [5].

For multi-sensor information fusion techniques, the Kalman filter is the most widespread and well known. It began to be used in the early 1960s for aerospace and military applications such as guidance, navigation and control systems, and is used in an extremely wide range of systems and equipment in almost all areas of engineering. Kalman filters can be divided into two main categories. The first category is the centralized Kalman filter (CKF) [6], where all measured sensor data are sent to a central site for processing. The advantage of this method is the minimal information dropout. However, it can lead to serious computational problems, which it can’t handle due to the filters being overloaded with more data. Therefore, when serious data failures occur, the entire centralized filter may become unreliable or have poor accuracy and stability. The second category is the distributed Kalman filter (DKF). Local estimators from all sensors can obtain a globally optimal or suboptimal state estimator according to certain information fusion criteria. The advantage of this method is that it no longer needs a fusion center that requires a large amount of memory space. Thus, in recent decades, various distributed and parallel versions and applications of the Kalman filter have been reported, such as in [7,8,9], to improve its accuracy. Hashmipour et al. [7] described a parallel Kalman filtering structure for multisensory networks amenable to parallel processing. Carlson [8] presented the famous federated square root filter, which assumes the initial estimation error cross covariance matrices among the local subsystems to be zero, i.e., the local estimation errors among the local subsystems are uncorrelated at the initial time, which does not accord with the general case. Ogle et al. [9], in turn, described a multi-sensor optimal information fusion estimator in the maximum likelihood sense under the assumption of normal distributions.

However, all of the above literature assumes that process noise and measurement noise are uncorrelated at any given moment. However, in actual sensor application environments, due to the use environment of sensor network, as well as its own factors, there is always related measurement noise and estimation noise. As a result, cross-correlation noise can be found in many engineering applications [10]. For example, the correlated noise in a discretized continuous-time system from [11]. Singular systems can transform into normal systems with associated noise, as described in [12]. The network system with random transmission delays [13] and packet dropout [14] can be transformed into a finite element system with step-like correlated noise. Signals measured or transmitted in a common noise environment usually have associated noise, and so on. However, the problem of reducing the impact of excessive covariance on the sensor system while achieving optimal estimation has not been solved yet. Since there is no matrix weight fusion method incorporating a feedback structure described in the previous literature, we consider whether a matrix weight fusion method with a feedback structure could be used to solve the mutual correlation problem between the multi-sensor measurement noise and the estimated noise, as well as to reduce the covariance generated during the sensor data fusion.

In addition, in UAV navigation and positioning, the sensor network of the UAV inevitably generates data packet dropout during transmission, in addition to measurement noise- and estimation noise-related situations. Therefore, in this case, the traditional Kalman filter is no longer applicable. To date, a variety of filtering approaches have been developed for systems with random time delay and multiple packet dropouts, including optimal full-order and reduced-order filters in the sense of linear minimum variance computed using the completion level method [15]. An optimal linear estimator in a unified model with random one-step sensor delay, multiple packet dropouts, and uncertain observations was described in [16], and adaptive filtering with a similar model to that described in [15,16] with an optimal linear estimator was described in [17]. Moreover, with the development of transmission through UAV sensor networks, the consideration of both network data packet dropout and correlated noise [18,19,20] has become a hot area of current research. A suboptimal Kalman-type filter was designed in [18] for systems with multi-packet dropout and finite step autocorrelation measurement noise. For the same system model, the optimal linear filter [19] was used to propose a minimum mean square error sense with better accuracy than [18]. For describing subsystems with packet dropout and correlated noise, filters, predictors and smoothers were also described in [20]. However, the impact of information packet dropout on the fusion results has not been deeply dissected under the optimal estimated fusion architecture with feedback. 

Based on the above discussion of the literature, this paper focuses on the problem of noise correlation for information fusion in distributed sensor networks in UAVs and the problem of packet dropout compensation during transmission. Different from as described in [18,19,20], in this paper, we use its predictive estimation as an optimal compensator. The proposed estimator based on predictive compensation has better accuracy than the estimator that uses simple compensation of the latest previously received measurements. Moreover, we use a filter with a feedback structure based on this, making it possible to reduce the covariance of each local tracking error while maintaining the optimality of the trajectory fusion. In summary, the main contributions of this paper are as follows:

(1) To address the unresolved issue of the impact of excessive covariance on sensor systems in [9,11,13], this paper proposes a matrix weight fusion method with a feedback structure. The method is not only able to deal with the problem of the inter-correlation of measurement and estimation noise in the process of multi-sensor data fusion, is can also achieve optimal estimation in a linear minimum variance sense.
(2) Based on the matrix weight fusion method with feedback structure proposed in this paper, the problem of the impact of information packet dropout on fusion results during sensor data transmission was not profiled in depth in [14,15,16]. This paper proposes an estimation and compensation method with a feedback structure; this method can reduce the covariance generated during the fusion of multi-sensor information.
(3) Finally, a Kalman smoothing algorithm is added to optimize the results of the Kalman filter fusion by forward and backward filtering to give better accuracy.

The rest of this paper is organized as follows: In Section 2, the studied problem is formulated. In Section 3, the optimal linear estimators with feedback, including the filter and the predictor, are designed. In Section 4, the optimal information fusion criterion in the linear minimum variance sense is provided. In Section 5, the Kalman optimal smoother linear estimators are given. In Section 6, a tracking example is reported. The last Section 7 of this paper provides the conclusion. 

## 2. Problem Formulation

In this paper, considering the following discrete-time stochastic systems with correlated noise and multiple packet dropouts for UAV sensor networks:

(1) $$x_{i}(t+1)=\Phi x_{i}(t)+\Gamma w_{i}(t)$$

(2) $$z_{i}(t)=Hx_{i}(t)+v_{i}(t)$$

(3) $$y_{i}(t)=\xi (t)z_{i}(t)+(1-\xi (t))z_{i}(t|t-1)$$

where $x_{i}(t)\in R^{n}$, *i* = 1, 2, 3,…, *l*, is the state, $z_{i}(t)\in R^{m_{i}}$ is the measurement value to be transmitted to the data processing center through the UAV sensor network, and $y_{i}(t)\in R^{m_{i}}$ is the measurement result received by the data processing center. Φ,Γ,H are time-varying matrices with compatible dimensions. A sequence of independent Bernoulli distribution variables {ξ(t)∈R} with probability [ξ(t)=1]=β(t), 0<β(t)≤1 is used to describe the packet dropout situation. This is not correlated with other random variables. From (3), it can be concluded that the measured value $z_{i}(t)$ of the sensor is received when ξ(t)=1 and is lost when ξ(t)=0. If the measurement result $z_{i}(t)$ is lost, its predictor $z_{i}(t|t-1)$ is used as a compensator, relying on the previously received information.

**Assumption** **1.** *w_i_(t) and v_i_(t), i = 1, 2, …, l are correlated white noises with zero mean and*

$$\begin{array}{l} E\left\{\left[\begin{array}{c} w_{i}(t)\\ v_{i}(t) \end{array}\left[\begin{array}{cc} w_{i}{}^{T}(k) & v_{i}^{T}(k) \end{array}\right]\right]\right\}=\left[\begin{array}{cc} Q_{i}(t) & S_{i}(t)\\ S_{i}^{T}(t) & R_{i}(t) \end{array}\right]\delta_{tk}\\ E\left[v_{i}(t)v_{j}^{T}(k)\right]=S_{ij}(t)\delta_{tk},\begin{array}{cc} & i\neq j \end{array} \end{array}$$

*where the symbol E denotes the mathematical expectation, the superscript T indicates the transpose, and* $\delta_{tk}$ *is the Kronecker delta function.*

**Assumption** **2.** *The initial state x(0) is independent of w_i_(t) and v_i_(t),i = 1, 2, …, l, and*

$$Ex(0)=\mu_{0},E\left[(x(0)-\mu_{0}){\left(x(0)-\mu_{0}\right)}^{T}\right]=P_{0}$$

**Assumption** **3.** *The optimal matrix weights,* ${\bar{A}}_{i}(t)$ *, i = 1, 2, …, l, to minimize the trace of the fusion filtering error variance,* $x(t|t)={\bar{A}}_{1}x_{1}(t|t)+{\bar{A}}_{2}x_{2}(t|t)+\ldots +{\bar{A}}_{l}x_{l}(t|t)$ *, where* ${\bar{A}}_{i}(t)$ *, i = 1, 2, …, l, are the weights, and* $x_{i}(t)$ *, i = 1, 2, …, l, are the local filters.*

**Assumption** **4.** *When there is feedback, the fusion center broadcasts its latest estimate to the local sensors. Thus, for all i, the following local state* $x_{i}(t)$* is replaced by multi-sensor fused state *x(t)*. The equation of state, Equation (1), can be rewritten as*

(4) $$x_{i}(t+1)=\Phi x(t)+\Gamma w_{i}(t)$$

Given a UAV sensor network, the communication topology among sensors is described by an undirected graph, G=(V,E,A), which consists of a node set V={1,2,…,n}, an edge set E⊆V×V. For an undirected graph G,(*i*, *j*) ∈E⟺(*j*,*i*) ∈E, that is, nodes *i* and *j* can sense each other. Graph G characterizes the communication topology among sensors and is connected if there exists a path involving all nodes.

## 3. Optimal Matrix Weight Fusion Kalman Filter with Feedback Structure

Under Assumptions 1 and 2, for the *i*-th local sensor subsystem of Systems (1)–(3), a local optimal Kalman filter with multiple sensors is proposed, as described below:

(5) $$x_{i}(t|t)=x_{i}(t|t-1)+K_{x_{i}}(t|t)\varepsilon_{i}(t)$$

(6) $$x_{i}(t+1|t)=\Phi x_{i}(t|t)+\Gamma w_{i}(t|t)$$

The innovation $\varepsilon_{i}(t)$ and the covariance matrix $Q_{\varepsilon_{i}}(t)$ can be obtained as

(7) $$\varepsilon_{i}(t)=z_{i}(t)-Hx_{i}(t|t-1)-v_{i}(t|t-1)$$

(8) $$Q_{\varepsilon_{i}}(t)=HP_{x_{i}}(t|t-1)H^{T}+P_{v_{i}}(t|t-1)$$

where the gain matrix $K_{x_{i}}(t|t)$ can be obtained by

(9) $$K_{x_{i}}(t/t)\begin{array}{c} = \end{array}[P_{x_{i}}(t/t-1)H^{T}]Q_{\varepsilon_{i}}^{-1}(t)$$

The estimated error covariance matrix $P_{x_{i}}(t/t)$ and the predictor $P_{x_{i}}(t+1/t)$ are given by

(10) $$P_{x_{i}}(t|t)=P_{x_{i}}(t|t-1)-K_{x_{i}}(t|t)Q_{\varepsilon_{i}}(t)K_{x_{i}}^{T}(t|t)$$

(11) $$P_{x_{i}}(t+1|t)=\Phi P_{x_{i}}(t|t)\Phi^{T}+\Gamma P_{w_{i}}(t|t)\Gamma^{T}$$

To obtain fusion results with higher accuracy, the local filtered values obtained above are fused with matrix weights.

**Lemma** **1.** *Under Assumptions 1 and 2, we can obtain the optimal fusion in a unified form* [8] *for distributed Kalman filters as*

(12) $$x={\bar{A}}_{1}x_{1}+{\bar{A}}_{2}x_{2}+\ldots +{\bar{A}}_{l}x_{l}$$

*where* $x_{i}$*, i = 1, 2, …, l, are the unbiased estimators of n-dimensional stochastic vector x. In addition, the optimal matrix weights* ${\bar{A}}_{i}$*, i = 1, 2, …, l are calculated by*

(13) $$\bar{A}=\Sigma^{-1}e{\left(e^{T}\Sigma^{-1}e\right)}^{-1}$$

*where* $\Sigma =P_{ij}$ *,* i,j=1,2…l *is an* nl×nl *symmetric positive definite matrix,* $\bar{A}={\left[{\bar{A}}_{1},{\bar{A}}_{2},\ldots ,{\bar{A}}_{l}\right]}^{T}$* and* $e={\left[I_{n},\ldots ,I_{n}\right]}^{T}$* are both* nl×n *matrices. The corresponding variance of the optimal information fusion estimator can be obtained by*

(14) $$P_{x}={\left(e^{T}\Sigma^{-1}e\right)}^{-1}$$

**Lemma** **2.** *The local Kalman filtering error cross covariance between the i-th and the j-th sensor subsystems has the following recursive form, from* [11]:

(15) $$\begin{array}{ll} P_{x_{i}x_{j}}(t+1|t+1) & =[I_{n}-K_{x_{i}}(t+1|t+1)H]\times \{\Phi P_{x_{i}x_{j}}(t|t)\Phi_{}^{T}+E[w_{i}(t|t)w_{j}^{T}(t|t)]\\ & +\Phi E[{\tilde{x}}_{i}(t|t)w_{j}^{T}(t|t)]+E[w_{i}(t|t){\tilde{x}}_{j}^{T}(t|t)]\Phi (t)\}\times [I_{n}-K_{x_{j}}(t+1|t+1)\\ & \times {H]}^{T}+HS_{ij}(t+1|t+1)K_{x_{j}}^{T}(t+1|t+1) \end{array}$$

*where* $P_{x_{i}x_{j}}$*, i, j = 1, 2, …, l are the filtering error cross covariance matrices between the i-th and j-th sensor subsystems,* $K_{x_{i}}$*,* $K_{x_{j}}$ *is the filtering gain matrix, and the initial values* $P_{x_{i}x_{j}}(0|0)=P_{x_{0}}$.

Next, we will show that the inclusion of a feedback structure reduces the covariance of the multi-sensor in the transmission process when optimal estimates are obtained.

**Theorem** **1.** *When feedback exists, under Assumption 4, the optimal estimation at the previous moment is regarded as the prior value at the next moment, then*

(16) $$\begin{array}{ll} x_{i}(t+1|t) & =\Phi x_{i}(t|t)+\Gamma w_{i}(t|t)\\ & =\Phi x(t|t)+\Gamma w_{i}(t|t)\\ & =\Phi x_{f}(t|t)+\Gamma w_{i}(t|t) \end{array}$$

(17) $$\begin{array}{ll} P_{x_{i}}(t+1|t) & =\Phi P_{x_{i}}(t|t)\Phi^{T}+\Gamma P_{w_{i}}(t|t)\Gamma^{T}\\ & =\Phi P_{x}(t|t)\Phi^{T}+\Gamma P_{w_{i}}(t|t)\Gamma^{T}\\ & =\Phi P_{x_{f}}(t|t)\Phi^{T}+\Gamma P_{w_{i}}(t|t)\Gamma^{T} \end{array}$$

*where the subscript “f” denotes the corresponding quantities in the feedback case. *x(t|t)* and *$P_{x}(t|t)$* can be obtained from Lemmas 1 and 2. In addition, it can be concluded that the trajectory fusion formula with feedback is the same as the trajectory fusion without feedback and the estimation error covariance matrices* $P_{x_{i}}^{}(t+1/t+1)\geq P_{x_{if}}^{}(t+1/t+1)$.

**Proof.**  After adding feedback, the predicted value of each sensor is as follows:

(18) $$\begin{array}{ll} x_{if}(t+1|t) & =\Phi x_{f}(t|t)+\Gamma w_{i}(t|t)\\ & =\Phi x_{f}(t+1|t) \end{array}$$

(19) $$\begin{array}{ll} P_{x_{if}}(t+1|t) & =\Phi P_{x_{f}}(t|t)\Phi^{T}+\Gamma P_{w_{i}}(t|t)\Gamma^{T}\\ & =P_{x_{f}}(t+1|t) \end{array}$$

(20) $$P_{x_{i}}(t+1|t+1)=P_{x_{i}}(t+1|t)-K_{x_{i}}(t+1|t+1)Q_{\varepsilon_{i}}(t+1)K_{x_{i}}^{T}(t+1|t+1)$$

Local covariance after adding feedback:

(21) $$\begin{array}{ll} P_{x_{if}}(t+1|t+1) & =P_{x_{i}}(t+1|t)-K_{x_{i}}(t+1|t+1)Q_{\varepsilon_{i}}(t+1)K_{x_{i}}^{T}(t+1|t+1)\\ & =P_{x_{if}}(t+1|t)-K_{x_{i}}(t+1|t+1)Q_{\varepsilon_{i}}(t+1)K_{x_{i}}^{T}(t+1|t+1)\\ & =P_{x_{f}}(t+1|t)-K_{x_{i}}(t+1|t+1)Q_{\varepsilon_{i}}(t+1)K_{x_{i}}^{T}(t+1|t+1) \end{array}$$

(20) and (21) are subtracted after each inverse matrix, giving us

(22) $$\begin{array}{l} P_{x_{i}}^{-1}(t+1|t+1)-P_{x_{if}}^{-1}(t+1|t+1)\\ =P_{x_{i}}^{-1}(t+1|t)-P_{x_{f}}^{-1}(t+1|t)\\ =P_{x_{i}}^{-1}(t+1|t)-P_{x}^{-1}(t+1|t)\\ ={\left[\Phi P_{x_{i}}(t|t)\Phi^{T}+\Gamma P_{w_{i}}(t|t)\Gamma \right]}^{-1}-{\left[\Phi P_{x}(t|t)\Phi^{T}+\Gamma P_{w_{i}}(t|t)\Gamma \right]}^{-1} \end{array}$$

(23) $$\begin{array}{l} P_{x}(t|t)={\left(e^{T}\Sigma^{-1}e\right)}^{-1}\\ ={\left[{\left(\Sigma^{-1/2}e\right)}^{T}(\Sigma^{1/2}e_{i})\right]}^{T}\times {\left[{\left(\Sigma^{-1/2}e\right)}^{T}(\Sigma^{-1/2}e)\right]}^{-1}[{\left(\Sigma^{-1/2}e\right)}^{T}(\Sigma^{1/2}e_{i})]\\ \leq {\left(\Sigma^{1/2}e_{i}\right)}^{T}(\Sigma^{1/2}e_{i})=P_{x_{i}}(t|t) \end{array}$$

(24) $$P_{x_{i}}^{-1}(t+1|t+1)\leq P_{x_{if}}^{-1}(t+1|t+1)$$

(25) $$P_{x_{i}}^{}(t+1|t+1)\geq P_{x_{if}}^{}(t+1|t+1)$$

Next

(26) $$\begin{array}{l} {P_{x_{if}}}^{-1}(t/t)x_{if}(t/t)\\ =P_{x_{if}}{}^{-1}(t/t)x(t/t-1)+H^{T}P_{v_{i}}{}^{-1}(t)(z_{i}(t)-Hx(t/t-1))\\ =P_{x_{if}}{}^{-1}(t/t)x(t/t-1)+H^{T}P_{v_{i}}{}^{-1}(t)z_{i}(t)-H^{T}P_{v_{i}}{}^{-1}(t)Hx(t/t-1)\\ =P_{x_{if}}{}^{-1}(t/t)x(t/t-1)+H^{T}P_{v_{i}}{}^{-1}(t)z_{i}(t)-P_{x_{if}}{}^{-1}(t/t)x(t/t-1)+\\ \;\;P_{x_{i}}{}^{-1}(t/t-1)x(t/t-1)\\ =H_{}{}^{T}P_{v_{i}}{}^{-1}(t)z_{i}(t)+P_{x_{i}}{}^{-1}(t/t-1)x(t/t-1) \end{array}$$

(27) $$\begin{array}{ll} P_{x}{}^{-1}(t/t)x(t/t) & =H^{T}P_{v_{i}}{}^{-1}(t)z_{i}(t)+P_{x}{}^{-1}(t/t-1)x(t/t-1)\\ & =\sum_{i=1}^{l}H^{T}(t)P_{v_{i}}{}_{}^{-1}(t)z_{i}(t)+P_{x}{}^{-1}(t/t-1)x(t/t-1) \end{array}$$

Trajectory fusion with feedback is expressed as

(28) $$\begin{array}{ll} P_{x_{f}}{}^{-1}(t/t) & =P_{x_{f}}{}^{-1}(t/t-1)+\sum_{i=1}^{l}(P_{x_{if}}{}^{-1}(t/t)-P_{x_{f}}{}^{-1}(t/t-1))\\ & =\sum_{i=1}^{l}P_{x_{if}}{}^{-1}(t/t)-(l-1)P_{x_{f}}{}^{-1}(t/t-1) \end{array}$$

(29) $$\begin{array}{ll} P_{x_{f}}{}^{-1}(t/t)x_{f}(t/t) & =P_{f}{}^{-1}(t/t-1)x_{f}(t/t-1)+\\ & \sum_{i=1}^{l}\left(P_{x_{if}}^{-1}(t/t)x_{if}(t/t)-P_{x_{f}}{}^{-1}(t/t-1)x_{f}(t/t-1\right)\\ & =\sum_{i=1}^{l}P_{x_{if}}^{-1}(t/t)x_{if}(t/t)-(l-1)P_{x_{f}}{}^{-1}(t/t-1)x_{f}(t/t-1)\\ & =\sum_{i=1}^{l}H^{T}P_{v_{i}}^{-1}(t)z_{i}(t)+P_{x}{}^{-1}(t/t-1)x(t/t-1) \end{array}$$

Then

(30) $$\begin{array}{ll} x_{f}(t/t) & =P_{x}(t/t)[P_{x}{}^{-1}(t/t-1)x(t/t-1)+\sum_{i=1}^{l}H_{}^{T}P_{v_{i}}^{-1}(t)z_{i}(t)]\\ & =x(t/t) \end{array}$$

This proof is completed. From the above proof, it can be obtained that feedback will not affect the global tracking performance at the fusion center, but can reduce the covariance of local sensor, so the feedback improves the local tracking performance of the sensor network. □

## 4. Optimal Linear Estimators with Dropout

In this section, we will use the prediction estimate as the optimal compensator from Systems (1)–(3) and derive the predicted gain matrix, noise intercorrelation and autocorrelation covariance matrix, measurement noise, and estimated noise required for the information fusion process when there is information packet dropout in Section 4.1, and with a linear optimal filter in Section 4.2.

### 4.1. Preliminary Lemmas

**Theorem** **2.** *Under Assumptions 1 and 2 and Systems (1)–(3), the innovation* $\varepsilon_{i}(t)$* and its covariance matrix* $Q_{\varepsilon_{i}}(t)$* are calculated by*

(31) $$\varepsilon_{i}(t)=y_{i}(t)-Hx_{i}(t|t-1)-v_{i}(t|t-1)$$

(32) $$Q_{\varepsilon_{i}}(t)=\beta (t)[HP_{x_{i}}(t|t-1)H^{T}+P_{v_{i}}(t|t-1)]$$

*where *$y_{i}(t)$* can be obtained from (3) with *$z_{i}(t|t-1)=Hx_{i}(t|t-1)+v_{i}(t|t-1)$.* The predictor state *$x_{i}(t|t-1)$* can be calculated by following (5), and its prediction error covariance matrix *$P_{x_{i}}(t|t-1)$* can be obtained from (11). *β(t)* is used to illustrate the packet dropout phenomenon. The process noise *$w_{i}(t|t)$* and the predictor of the measurement noise *$v_{i}(t|t-1)$* are both calculated using Theorem 3. The covariance matrices *$P_{v_{i}}(t|t-1)$* can be calculated by following Theorem 4.*

**Proof.**  From the projection theorem in [21], the innovation $\varepsilon_{i}(t)=y_{i}(t)-y_{i}(t|t-1)$ can be obtained. Each term on both sides of (3) is projected onto the linear space generated by the measured value $\left(y_{i}(0),\;y_{i}(1),\;\ldots ,\;y_{i}(t-1)\right)$, giving us $y_{i}(t|t-1)=Hx_{i}(t|t-1)+v_{i}(t|t-1)$. Then, (31) can be obtained. Additionally, the innovation can be rewritten as follows using (2) and (3):

(33) $$\varepsilon_{i}(t)=\xi (t)[H{\tilde{x}}_{i}(t|t-1)+{\tilde{v}}_{i}(t|t-1)]$$

Substituting (33) into the covariance matrix $Q_{\varepsilon_{i}}(t)=E[\varepsilon_{i}(t)\varepsilon_{{}^{i}}^{T}(t)]$, we can obtain

(34) $$\begin{array}{ll} Q_{\varepsilon_{i}}(t) & =E[\varepsilon_{i}(t)\varepsilon_{{}^{i}}^{T}(t)]\\ & =\beta (t)\{HP_{x_{i}}(t|t-1)H^{T}+P_{v_{i}}(t|t-1)+HE[{\tilde{x}}_{i}(t|t-1){\tilde{v}}_{i}^{T}(t|t-1)]\\ & +E[{\tilde{v}}_{i}(t|t-1){\tilde{x}}_{i}^{T}(t|t-1)]H^{T} \end{array}$$

From (34) and $E[{\tilde{x}}_{i}(t|t-1){\tilde{v}}_{i}^{T}(t|t-1)]=0$, (32) can be obtained. This proof is completed. □

**Theorem** **3.** *Under Assumptions 1 and 2 and Systems (1)–(3), the process noise* $w_{i}(t|t)$ *and its gain matrix* $K_{w_{i}}(t|t-\tau )$ *are calculated by*

(35) $$w_{i}(t/t)=\sum_{\tau =0}^{N_{t}}K_{w_{i}}(t|t-\tau )\varepsilon_{i}(t-\tau )$$

(36) $$\begin{array}{ll} K_{w_{i}}(t|t-\tau ) & =\beta (t-\tau )[P_{w_{i}x_{i}}(t,t-\tau |t-\tau -1)H^{T}\\ & +P_{w_{i}v_{i}}(t,t-\tau |t-\tau -1)]Q_{\varepsilon_{i}}^{-1}(t-\tau )\\ & \beta (t-\tau )P_{w_{i}v_{i}}(t,t-\tau |t-\tau -1)Q_{\varepsilon_{i}}^{-1}(t-\tau ) \end{array}$$

The predictor of the measurement noise $v_{i}(t|t-1)$ and its gain matrix $K_{v_{i}}(t|t-\tau )$ are calculated by

(37) $$v_{i}(t|t-1)=\sum_{\tau =1}^{N_{t}}K_{v_{i}}(t|t-\tau )\varepsilon_{i}(t-\tau ),\;t\geq 1$$

(38) $$K_{v_{i}}(t|t-\tau )=\beta (t-\tau )P_{v_{i}}(t,t-\tau |t-\tau -1)Q_{\varepsilon_{i}}^{-1}(t-\tau ),\;t\geq \tau$$

where $v_{i}(0|-1)=0$, the covariance matrices $P_{w_{i}v_{i}}(t,t-\tau |t-\tau -1)$ and $P_{v_{i}}(t,t-\tau |t-\tau -1)$ are calculated following Theorem 3.

**Proof.**  From the projection theorem in [21], we obtain

(39) $$v_{i}(t|t)=v_{i}(t|t-N_{t}-1)+\sum_{\tau =1}^{N_{t}}K_{v_{i}}(t|t-\tau )\varepsilon_{i}(t-\tau )$$

which yields the measurement noise filter (37) by noting $v_{i}(t|t-N_{t}-1)=0$ and *N_t_* = min{*t*,*N*}. Using (33), the process noise in the τ-step prediction gain matrix is calculated by:

(40) $$\begin{array}{ll} K_{v_{i}}(t/t-\tau ) & =E[v_{i}(t)\varepsilon_{{}^{i}}^{T}(t-\tau )]Q_{\varepsilon_{i}}^{-1}(t-\tau )\\ & =\beta (t-\tau )E\{v_{i}^{}(t)[H{\tilde{x}}_{i}(t-\tau /t-\tau -1)\\ & +{\tilde{v}}_{i}(t-\tau /t-\tau -1){]}^{T}\}Q_{\varepsilon_{i}}^{-1}(t-\tau ) \end{array}$$

From (40), and noting $\begin{array}{c} E[v_{i}^{}(t){\tilde{x}}_{i}^{T}(t-\tau |t-\tau -1) \end{array}]=P_{w_{i}x}{}_{i}(t,t-\tau |t-\tau -1)=0$ and $\begin{array}{c} E[v_{i}^{}(t){\tilde{v}}_{i}^{T}(t-\tau |t-\tau -1) \end{array}]=P_{v_{i}}(t,t-\tau |t-\tau -1)$, we obtain (38). Similarly, (35) and (36) can be derived. This proof is completed. □

**Theorem** **4.** *For Systems (1)–(3) under Assumptions 1 and 2, the following covariance matrices can be obtained:*

*The cross covariance matrix of the estimation error of the process noise *$P_{w_{i}}(t,t-k|t-k)$* and the cross covariance matrix of the estimation error of the measurement noise *$P_{v_{i}}(t,t-k|t-k)$ *are calculated by*

(41) $$P_{w_{i}}(t,t-k|t-k)=Q(k)-\sum_{l=k+1}^{N_{t}}K_{w_{i}}(t|t-l)Q_{\varepsilon_{i}}(t-l)K_{w_{i}}^{T}(t-k|t-l)$$

(42) $$P_{v_{i}}(t,t-k|t-k-1)=R_{i}(k)-\sum_{l=k+1}^{N_{t}}K_{v_{i}}(t|t-l)Q_{\varepsilon_{i}}(t-l)K_{v_{i}}^{T}(t-k|t-l)$$

*where *t≥k+1* and *$P_{v_{i}}(k,0|-1)=R_{i}(k)$. *The estimation error cross covariance matrix *$P_{w_{i}v_{i}}(t,t-k/t-k-1)$* between the process noise and the measurement noise is calculated as*

(43) $$P_{w_{i}v_{i}}(t,t-k|t-k-1)=S_{i}(k)-\sum_{l=k+1}^{N_{t}}K_{w_{i}}(t|t-l)Q_{\varepsilon_{i}}(t-l)K_{v_{i}}^{T}(t-k|t-l)$$

*where *$t\geq k+1,\;P_{w_{i}v_{i}}(k,0|-1)=S_{i}\left(k\right)$.

**Proof.**  Subtracting (35) from $w_{i}(t)$, the filtering error equation of the process noise can be obtained:

(44) $${\tilde{w}}_{i}(t/t)=w_{i}(t)-\sum_{\tau =0}^{N_{t}}K_{w_{i}}(t/t-\tau )\varepsilon_{i}(t-\tau )$$

Substituting (44) into $P_{w_{i}}(t,t-k/t-k)=E[w_{i}(t){\tilde{w}}_{i}{}^{T}(t-k/t-k)]$, we can obtain (41).

Substituting the above (37) into $v_{i}(t)$, the measurement noise ${\tilde{v}}_{i}(t|t-1)$ can be obtained:

(45) $${\tilde{v}}_{i}(t|t-1)=v_{i}(t)-\sum_{\tau =1}^{N_{t}}K_{v_{i}}(t|t-\tau )\varepsilon_{i}(t-\tau )$$

Substituting (45) into $P_{w_{i}v_{i}}(t,t-k|t-k-1)=E[w_{i}(t){\tilde{v}}_{i}^{T}(t-k|t-k-1)]$, we get (43). Similarly, using (45) into $P_{v_{i}}(t,t-k/t-k)=E[v_{i}(t){\tilde{v}}_{i}{}^{T}(t-k/t-k)]$, we can derive (42). This proof is completed. With the above proof, we derive the predicted gain matrix, noise intercorrelation and autocorrelation covariance matrix, measurement noise and estimated noise required for the information fusion process when the measurement information drops packets. □

### 4.2. Linear Optimal Filter with Feedback

**Theorem** **5.** *Under Assumptions 1 and 2 and Systems (1)–(3), the optimal linear local filter and local predictor are calculated by:*

(46) $$x_{i}(t|t)=x_{i}(t|t-1)+K_{x_{i}}^{d}(t|t)\varepsilon_{i}(t)$$

(47) $$x_{i}(t+1|t)=\Phi x_{i}(t|t)+\Gamma w_{i}(t|t)$$

*The gain matrix* $K_{x_{i}}^{d}(t|t)$ *can be obtained as*

(48) $$K_{x_{i}}^{d}\begin{array}{c} = \end{array}\beta (t)[P_{x_{i}}(t/t-1)H^{T}]Q_{\varepsilon_{i}}^{-1}(t)$$

*The covariance matrix of state quantities* $P_{x_{i}}(t/t)$ *and the predictor* $P_{x_{i}}(t+1/t)$ *are calculated by*

(49) $$P_{x_{i}}(t|t)=P_{x_{i}}(t|t-1)-K_{x_{i}}^{d}(t|t)Q_{\varepsilon_{i}}(t){\left[K_{x_{i}}^{d}(t|t)\right]}^{T}$$

(50) $$P_{x_{i}}(t+1|t)=\Phi P_{x_{i}}(t|t)\Phi^{T}+\Gamma P_{w_{i}}(t|t)\Gamma^{T}$$

**Proof.**  According to the projection theorem from [21], we have (46), and the gain matrix $K_{x_{i}}^{d}$ is defined by

(51) $$K_{x_{i}}^{d}\begin{array}{c} = \end{array}E[x_{i}(t)\varepsilon_{i}^{T}(t)]Q_{\varepsilon_{i}}^{-1}(t)$$

Subtracting (33) from (51), we can obtain

(52) $$K_{x_{i}}^{d}\begin{array}{c} = \end{array}\beta (t)E\{x_{i}(t){\left[H{\tilde{x}}_{i}(t|t-1)+{\tilde{v}}_{i}(t|t-1)\right]}^{T}\}Q_{\varepsilon_{i}}^{-1}(t)$$

Note that ${\hat{x}}_{i}(t|t-1)⊥{\tilde{x}}_{i}(t|t-1)$ and ${\hat{x}}_{i}(t|t-1)⊥{\tilde{v}}_{i}(t|t-1)$, and we can obtain (48). From (46), the filtering error equation for the state can be obtained:

(53) $${\tilde{x}}_{i}(t|t)={\tilde{x}}_{i}(t|t-1)-K_{x_{i}}^{d}\varepsilon_{i}(t)$$

Substituting (53) into the filtering error covariance matrix $P_{x_{i}}(t|t)=E[{\tilde{x}}_{i}(t|t){\tilde{x}}_{i}^{T}(t|t)]$ and ${\hat{x}}_{i}(t|t-1)⊥\varepsilon_{i}(t)$, the following can be obtained:

(54) $$\begin{array}{ll} P_{x_{i}}(t|t) & =P_{x_{i}}(t|t-1)+K_{x_{i}}^{d}(t|t)Q_{\varepsilon_{i}}(t){\left[K_{x_{i}}^{d}(t|t)\right]}^{T}\\ & -E[x_{i}(t)\varepsilon_{i}^{T}(t)]{\left[K_{x_{i}}^{d}(t|t)\right]}^{T}-K_{x_{i}}^{d}(t|t)E[\varepsilon_{i}(t)x_{i}^{T}(t)] \end{array}$$

From (54), and noting $E[x_{i}(t)\varepsilon_{i}(t)]=K_{x_{i}}^{d}(t|t)Q_{\varepsilon_{i}}(t)$, we can obtain (49).

Subtracting (47) from (1), the predictor $x_{i}(t+1|t)$ can be obtained.

Substituting ${\tilde{x}}_{i}(t+1|t)=\Phi {\tilde{x}}_{i}(t|t)+\Gamma {\tilde{w}}_{i}(t|t)$ into the error covariance matrix $P_{x_{i}}(t+1|t)=E[{\tilde{x}}_{i}(t+1|t){\tilde{x}}_{i}^{T}(t+1|t)]$, it is possible to obtain (50). □

**Theorem** **6.** *When feedback exists, under Assumption 4, the predictor of the next moment can be same as (16), (17)*

(55) $$\begin{array}{ll} x_{i}(t+1|t) & =\Phi x_{i}(t|t)+\Gamma w_{i}(t|t)\\ & =\Phi x(t|t)+\Gamma w_{i}(t|t) \end{array}$$

(56) $$\begin{array}{ll} P_{x_{i}}(t+1|t) & =\Phi P_{x_{i}}(t|t)\Phi^{T}+\Gamma P_{w_{i}}(t|t)\Gamma^{T}\\ & +\Phi P_{x_{i}w}{}_{i}(t|t)\Gamma^{T}+\Gamma P_{x_{i}w_{i}}^{T}(t|t)\Phi^{T}\\ & =\Phi P_{x}(t|t)\Phi^{T}+\Gamma P_{w_{i}}(t|t)\Gamma^{T} \end{array}$$

Furthermore, it can already be concluded that the estimation error covariance matrices $P_{x_{i}}^{}(t+1/t+1)\geq P_{x_{if}}^{}(t+1/t+1)$.

**Proof.**  After adding feedback, the predicted value of each sensor is as follows

(57) $$\begin{array}{ll} x_{if}(t+1|t) & =\Phi x_{i}(t|t)+\Gamma w_{i}(t|t)\\ & =\Phi x_{f}(t|t)+\Gamma w_{i}(t|t)\\ & =\Phi x_{f}(t+1|t) \end{array}$$

(58) $$\begin{array}{ll} P_{x_{if}}(t+1|t) & =\Phi P_{x_{i}}(t|t)\Phi^{T}+\Gamma P_{w_{i}}(t|t)\Gamma^{T}\\ & =\Phi P_{x_{f}}(t|t)\Phi^{T}+\Gamma P_{w_{i}}(t|t)\Gamma^{T}\\ & =P_{x_{f}}(t+1|t) \end{array}$$

(59) $$\begin{array}{ll} P_{x_{i}}(t+1|t+1)= & P_{x_{i}}(t+1|t)-K_{x_{i}}^{d}(t+1|t+1)\\ & \times Q_{\varepsilon_{i}}(t+1){\left[K_{x_{i}}^{d}(t+1|t+1)\right]}^{T} \end{array}$$

Local covariance after adding feedback:

(60) $$\begin{array}{ll} P_{x_{if}}(t+1|t+1) & =P_{x_{i}}(t+1|t)-K_{x_{i}}^{d}(t+1|t+1)Q_{\varepsilon_{i}}(t+1){\left[K_{x_{i}}^{d}(t+1|t+1)\right]}^{T}\\ & =P_{x_{if}}(t+1|t)-K_{x_{i}}^{d}(t+1|t+1)Q_{\varepsilon_{i}}(t+1){\left[K_{x_{i}}^{d}(t+1|t+1)\right]}^{T}\\ & =P_{x_{f}}(t+1|t)-K_{x_{i}}^{d}(t+1|t+1)Q_{\varepsilon_{i}}(t+1){\left[K_{x_{i}}^{d}(t+1|t+1)\right]}^{T} \end{array}$$

It can be seen that (59) and (60) are the same as (20) and (21). Similarly, it can be concluded that $P_{x_{i}}^{}(t+1|t+1)\geq P_{x_{if}}^{}(t+1|t+1)$.

This proof is completed. □

## 5. Kalman Smoothing Algorithm

In this section, to further enhance the fusion effect, we will optimize the fusion result using a smoothing process, thereby proposing the Kalman smoothing algorithm.

**Theorem** **7.** *The smoothed parameters* $x_{s}(t|t)$ and $P_{s}(t|t)$ *can be written as*

(61) $$x_{s}(t|t)=x_{+}(t|t)+K_{-}(t|t)[x_{s}(t+1|t+1)-x_{-}(t+1|t)]$$

(62) $$P_{s}(t|t)=P_{+}(t|t)+K_{-}(t|t)[P_{s}(t+1|t+1)-P_{-}(t+1|t)]\times K_{-}^{T}(t|t)$$

$x_{+}(t|t)$ and $P_{+}(t|t)$ represent the fusion estimate of the posterior estimate of time *t*. $x_{-}(t+1|t)$ and $P_{-}(t+1|t)$ represent the fusion estimate of the prior estimate of time *t*. $K_{-}(t|t)$ represents the reverse filtering gain matrix.

The smoothing algorithm consists of forward filtering and backward filtering. The forward filtering consists of the classical Kalman filter, which is used to estimate the state at each moment. The backward filtering reuses some data on the basis of the forward filtering to obtain a more accurate state estimate.

**Proof.**  From (46)–(50), the local optimal Kalman filter can be obtained as

(63) $$x_{i}(t|t-1)=\Phi x_{i}(t-1|t-1)+\Gamma w_{i}(t-1|t-1)$$

(64) $$P_{x_{i}}(t|t-1)=\Phi P_{x}(t-1|t-1)\Phi^{T}+\Gamma P_{w_{i}}(t-1|t-1)\Gamma^{T}$$

The gain matrix of the forward recursion is

(65) $$\begin{array}{ll} K_{x_{i}}(t|t) & =\beta (t)[P_{x_{i}}(t|t-1)H^{T}+P_{v_{i}}(t|t-1)+P_{v_{i}x_{i}}^{T}(t|t-1)]Q_{\varepsilon_{i}}^{-1}(t)\\ & =\beta (t)[P_{x_{i}}(t|t-1)H^{T}]Q_{\varepsilon_{i}}^{-1}(t) \end{array}$$

The fusion estimate of the posterior estimate of time *t* is

(66) $$x_{+}(t|t)=x_{i}(t|t-1)+K_{x_{i}}(t|t)\varepsilon_{i}(t)$$

(67) $$P_{+}(t|t)=P_{x_{i}}(t|t-1)-K_{x_{i}}(t|t)Q_{\varepsilon_{i}}(t)K_{x_{i}}^{T}(t|t)$$

The Kalman filter forward recursion is

(68) $$x_{\_}(t+1|t)=\Phi x_{+}(t|t)+\Gamma {\hat{w}}_{i}(t|t)$$

(69) $$P_{\_}(t+1|t)=\Phi P_{+}(t|t)\Phi^{T}+\Gamma P_{w_{i}}(t|t)\Gamma^{T}$$

The gain matrix of the backward recursion is

(70) $$K_{\_}(t|t)=P_{+}(t|t)H^{T}{\left[P_{\_}(t+1|t)\right]}^{-1}$$

Therefore, the fusion estimation of reverse filtering is

(71) $$x_{s}(t|t)=x_{+}(t|t)+K_{-}(t|t)[x_{s}(t+1|t+1)-x_{-}(t+1|t)]$$

(72) $$\begin{array}{l} P_{s}(t|t)=P_{+}(t|t)+K_{-}(t|t)[P_{s}(t+1|t+1)-P_{-}(t+1|t)]\\ \begin{array}{cc} & \times \end{array}K_{-}^{T}(t|t) \end{array}$$

*t* forward recursion is performed from the initial time to time *t*, and then backward recursion is performed from time *t* to *t*. This completes the Kalman smoothing process. This proof is completed. □

## 6. Simulation Example

Consider a radar tracking system with three sensors:

(73) $$x_{i}(t+1)=\left[\begin{array}{ccc} 1 & T & T^{2}/2\\ 0 & 1 & T\\ 0 & 0 & 1 \end{array}\right]x_{i}(t)+\left[\begin{array}{c} 0\\ 0\\ 1 \end{array}\right]w_{i}(t)$$

(74) $$z_{i}(t)=Hx_{i}(t)+v_{i}(t)$$

(75) $$y_{i}(t)=\xi (t)z_{i}(t)+(1-\xi_{i}(t))z_{i}(t|t-1)$$

(76) $$v_{i}(t)=\gamma_{i}w(t)+\zeta_{i}(t)$$

i=1,2,3

where *T* is the sampling period. The state is $x(t)={\left[s(t)\dot{s}(t)\ddot{s}(t)\right]}^{T}$, where $s(t),\dot{s}(t),\ddot{s}(t)$ are the position, velocity and acceleration, respectively, of the target at time *t*, $y_{i}(t)$, *i* = 1, 2, 3 are the measurement signals, $v_{i}(t)$, *i* = 1, 2, 3 are the measurement noises, respectively, of three sensors, which are correlated with Gaussian white noise *w_i_*(*t*) with mean zero and variance $\sigma_{w}^{2}$. The coefficients $\gamma_{i}$ are constant scalars, and $\zeta_{i}(t)$, *i* = 1, 2, 3 are Gaussian white noises with means of zero and variance matrices $\sigma_{\zeta_{i}}^{2}$, and which are independent of *w_i_*(*t*). Our aim is to find the optimal information fusion decentralized Kalman filter ${\hat{x}}_{0}(t\left|t)\right.$.

We set T=0.01, $H_{1}=[1,0,0]$, $H_{2}=[0,1,0]$, $H_{3}=[0,0,1]$; $\sigma_{\omega }^{2}=1,\sigma_{\zeta_{1}}^{2}=5,\sigma_{\zeta_{2}}^{2}=8,\sigma_{\zeta_{3}}^{2}=8$; $\gamma_{1}=2,\gamma_{2}=1,\gamma_{3}=1$; and initial values $x(0)={\left[0,0,0\right]}^{T},P_{0}=0.1I_{3}$. For each sensor system, by applying (46)–(50), the local optimal Kalman filter $x_{i}(t/t)$ and corresponding variances $P_{x_{i}}(t/t)$, *i* = 1, 2, 3 can be obtained. Then, the optimal information fusion filter x(t|t) and corresponding variance $P_{x}(t|t)$ can be obtained from Lemma 1. Additionally, we substitute the obtained results x(t|t) and $P_{x}(t|t)$ into (55) and (56), allowing the predictor of the estimation error covariance matrices $P_{x_{i}}(t+1/t)$ and the optimal fusion $x_{i}(t+1|t)$ to be obtained.

The simulation is divided into two parts. Firstly, the tracking effects of distributed Kalman filtering and distributed Kalman filtering with dropout are compared in the case of the same target motion. Secondly, the tracking effect between the distributed Kalman filter with dropout, the distributed Kalman filter with dropout and feedback, and the smoothing algorithm are compared. Additionally, a comparison of the local covariance at different times is given in a table.

### 6.1. The Tracking Effects of Distributed Kalman Filtering with Dropout

**Figure 1:**
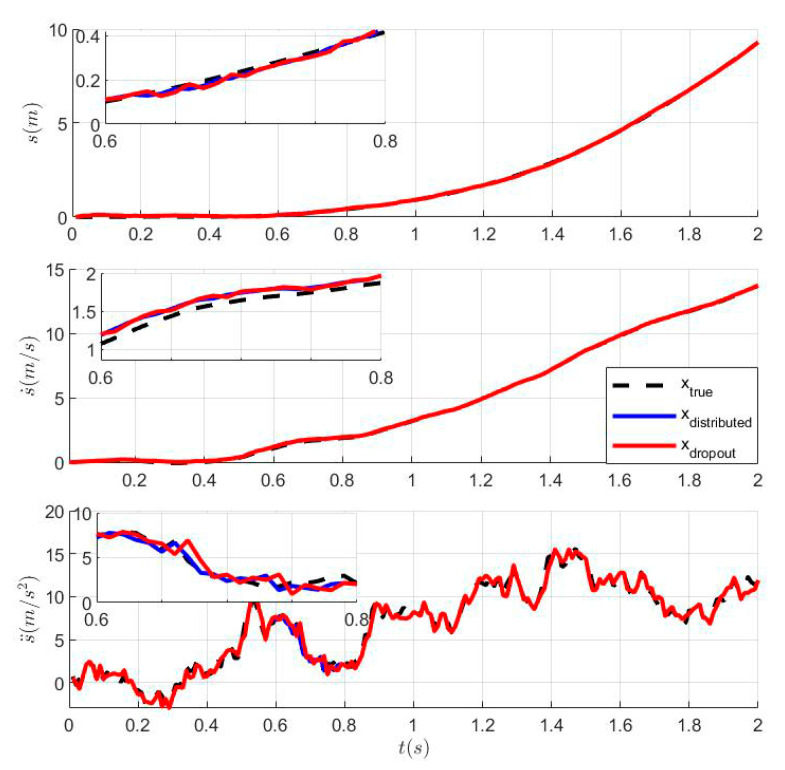
Comparison of the tracking effect between distributed Kalman filtering and distributed Kalman filtering with dropout.

**Figure 2:**
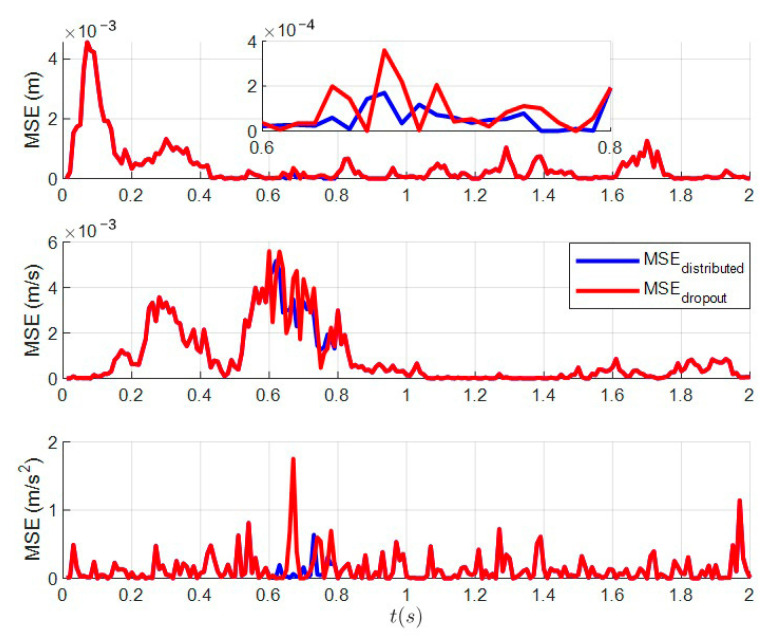
MSE of distributed Kalman filtering and distributed Kalman filtering with dropout.

Dropout during transmission is inevitable due to the performance differences of different sensors and external interference. Thus, it is assumed that dropout exists at *t* = 0.6–0.8 s and is compensated by the predicted value $z_{i}(t|t-1)$ of the previous moment. Figure 1 shows a comparison of the tracking effect between distributed Kalman filtering and distributed Kalman filtering with dropout, and Figure 2 shows the MSE of distributed Kalman filtering and distributed Kalman filtering with dropout. The presence of dropout at 0.6–0.8 s in Figure 2 is clearly indicated.

### 6.2. The Tracking Performance of Distributed Kalman Filtering with Dropout and Distributed Kalman Filtering with Dropout after Adding Feedback and the Smoothing Algorithm

**Figure 3:**
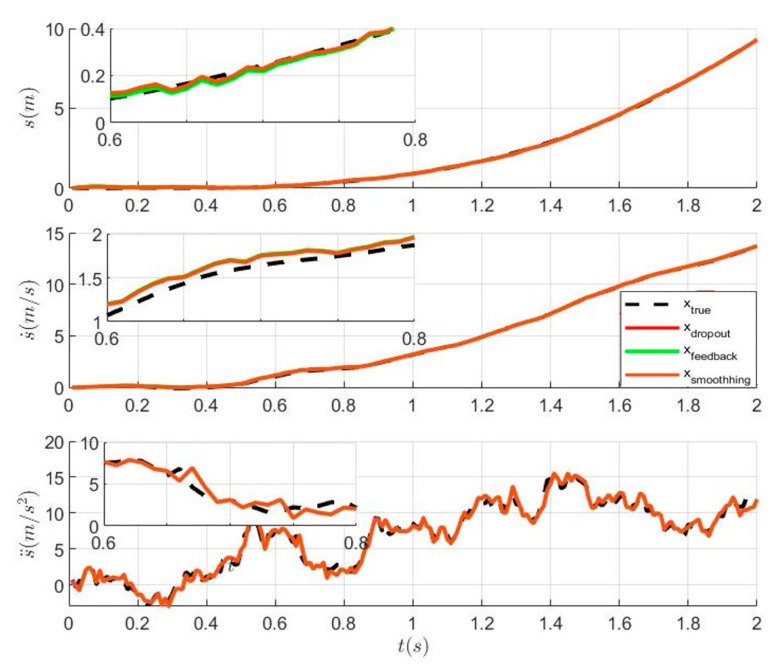
Comparison of the tracking effect between the distributed Kalman filter with dropout, feedback, and the smoothing algorithm.

**Figure 4:**
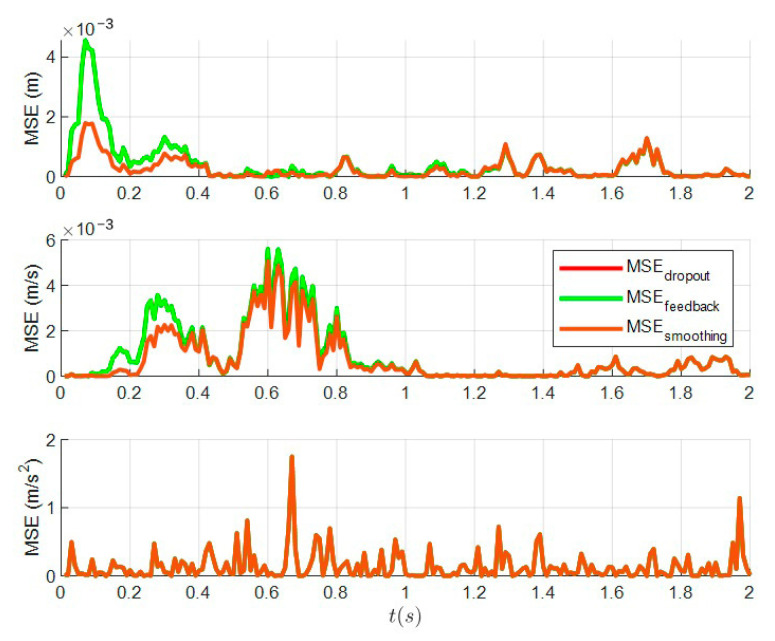
MSE of the distributed Kalman filter with dropout, feedback and the smoothing algorithm.

In this section, the tracking trajectories under four different conditions are reported. From Figure 3, it can be seen that the tracking performance of the distributed Kalman filter with feedback and the distributed Kalman filter without feedback are consistent when multiple sensors experience dropout during transmission. Therefore, this case confirms Theorem 4 in Section 3. Then, the tracking trajectory of the optimal fusion Kalman filter after adding the smoothing algorithm is close to the real value from Figure 3. This shows that the tracking effect of Kalman filter can be improved by adding the smoothing algorithm. A clearer comparison in Figure 4 shows that the addition of the smoothing algorithm can reduce filtering error.

Furthermore, we collected the optimal fusion covariance and local covariance for five different time nodes. $P_{a}$, $P_{b}$, $P_{c}$ represent respectively the error values of the local covariance with feedback and local covariance of the three sensors. It can be seen from Table 1 that the error value at each time point is consistent with the proof result in $P_{x_{i}}^{}(t+1|t+1)\geq P_{x_{if}}^{}(t+1|t+1)$.

## 7. Conclusions

In this paper, the correlation noise and packet dropout estimation problems of information fusion in distributed sensing networks are investigated. In contrast to previous studies, in this paper, a matrix weight fusion method is proposed in combination with a feedback structure to solve the problem of the correlation between measurement noise and estimation noise generated in sensor networks, effectively solving the mutual correlation problem between multi-sensor measurement noise and estimation noise while achieving optimal estimation in the sense of linear minimum variance. In addition, for the problem of packet dropout in the fusion process, a loss estimation compensation method with a feedback structure is proposed for the multi-sensor information fusion process, successfully reducing the covariance in the fusion process. Finally, the simulation shows that the MSE between the local covariance with the feedback structure and the local covariance without the feedback structure at the selected time node *t* is between 0 and 0.3, verifying that the local covariance with the feedback structure is smaller than the local covariance without the feedback structure, and thereby proving the effectiveness of the algorithm.

## Figures and Tables

**Table 1:** The error between the local covariance with feedback and the local covariance with date packet dropout at different times.

	*P*	$P_{a}$	$P_{b}$	$P_{c}$
*t*				
0.2		$\left[\begin{array}{ccc} 0.000 & 0.000 & 0.010\\ 0.000 & 0.020 & 0.009\\ 0.010 & 0.009 & 0.009 \end{array}\right]$	$\left[\begin{array}{ccc} 0.000 & 0.000 & 0.033\\ 0.000 & 0.010 & 0.011\\ 0.033 & 0.011 & 0.010 \end{array}\right]$	$\left[\begin{array}{ccc} 0.000 & 0.000 & 0.000\\ 0.000 & 0.008 & 0.005\\ 0.000 & 0.005 & 0.008 \end{array}\right]$
0.4		$\left[\begin{array}{ccc} 0.000 & 0.010 & 0.006\\ 0.010 & 0.018 & 0.010\\ 0.006 & 0.010 & 0.053 \end{array}\right]$	$\left[\begin{array}{ccc} 0.000 & 0.011 & 0.032\\ 0.011 & 0.010 & 0.008\\ 0.032 & 0.008 & 0.260 \end{array}\right]$	$\left[\begin{array}{ccc} 0.000 & 0.006 & 0.000\\ 0.006 & 0.008 & 0.001\\ 0.008 & 0.001 & 0.170 \end{array}\right]$
0.6		$\left[\begin{array}{ccc} 0.000 & 0.000 & 0.019\\ 0.000 & 0.005 & 0.006\\ 0.019 & 0.006 & 0.183 \end{array}\right]$	$\left[\begin{array}{ccc} 0.000 & 0.000 & 0.021\\ 0.000 & 0.023 & 0.021\\ 0.021 & 0.021 & 0.300 \end{array}\right]$	$\left[\begin{array}{ccc} 0.000 & 0.000 & 0.008\\ 0.000 & 0.006 & 0.008\\ 0.008 & 0.008 & 0.122 \end{array}\right]$
0.8		$\left[\begin{array}{ccc} 0.008 & 0.000 & 0.006\\ 0.000 & 0.008 & 0.009\\ 0.006 & 0.009 & 0.190 \end{array}\right]$	$\left[\begin{array}{ccc} 0.000 & 0.005 & 0.005\\ 0.005 & 0.023 & 0.005\\ 0.005 & 0.005 & 0.300 \end{array}\right]$	$\left[\begin{array}{ccc} 0.018 & 0.011 & 0.012\\ 0.011 & 0.008 & 0.008\\ 0.012 & 0.008 & 0.092 \end{array}\right]$
1.0		$\left[\begin{array}{ccc} 0.010 & 0.000 & 0.021\\ 0.000 & 0.010 & 0.011\\ 0.021 & 0.011 & 0.221 \end{array}\right]$	$\left[\begin{array}{ccc} 0.000 & 0.000 & 0.006\\ 0.000 & 0.008 & 0.005\\ 0.006 & 0.005 & 0.251 \end{array}\right]$	$\left[\begin{array}{ccc} 0.000 & 0.000 & 0.021\\ 0.000 & 0.014 & 0.013\\ 0.021 & 0.013 & 0.082 \end{array}\right]$

## Abbreviation

The following abbreviations are used in this manuscript:

UAV	unmanned aerial vehicle
KF	Kalman filter
CKF	centralized Kalman filter
DKF	distributed Kalman filter
MSE	mean square error
